# Fibroblast-like Synoviocytes as Therapeutic Targets in Rheumatoid Arthritis: Current Evidence on DMARD-Mediated Modulation

**DOI:** 10.3390/biomedicines14081784

**Published:** 2026-08-07

**Authors:** Sandra Pascual-García, Raúl Cobo, Pascual Martínez-Peinado, Alejandro Peco Mas, Lorena Ramos Gómez, José Miguel Sempere-Ortells

**Affiliations:** Department of Biotechnology, University of Alicante, 03690 San Vicente del Raspeig, Spain; sandra.pascual@ua.es (S.P.-G.);

**Keywords:** rheumatoid arthritis, fibroblast-like synoviocytes, DMARDs, Janus kinase inhibitors, bDMARDs, csDMARDs, precision medicine

## Abstract

**Background/Objectives**: Fibroblast-like synoviocytes (FLS) are key contributors to rheumatoid arthritis (RA) pathogenesis, driving synovial inflammation, cartilage degradation, bone erosion and disease persistence. Recent advances have revealed substantial FLS heterogeneity, with distinct fibroblast subsets exhibiting different pathogenic roles within the rheumatoid synovium. Although disease-modifying antirheumatic drugs (DMARDs) constitute the cornerstone of RA treatment, their effects on FLS have not been comprehensively characterised. This review summarises and compares the effects of conventional synthetic DMARDs (csDMARDs), biologic DMARDs (bDMARDs) and targeted synthetic DMARDs (tsDMARDs) on RA-FLS. **Methods**: A non-systematic literature review was conducted to identify studies investigating the effects of DMARDs on RA-FLS. Studies evaluating the impact of csDMARDs, bDMARDs and tsDMARDs on FLS proliferation, apoptosis, migration, invasion, inflammatory mediator production, extracellular matrix remodelling and osteoclastogenic activity were included. **Results**: Available evidence indicates that DMARDs modulate multiple pathogenic functions of RA-FLS. Methotrexate, leflunomide, hydroxychloroquine and sulfasalazine regulate inflammatory signalling, apoptosis, autophagy and ferroptosis. Biologic agents, particularly tumour necrosis factor alpha (TNF-α) and interleukin (IL)-6 receptor inhibitors, suppress cytokine production, matrix metalloproteinase expression, osteoclastogenic signalling and FLS migration. Targeted synthetic DMARDs, particularly Janus kinase inhibitors (JAKis), exhibit broad activity across inflammatory, angiogenic, metabolic and tissue-destructive pathways. Despite their distinct molecular targets, all DMARD classes ultimately attenuate key pathogenic FLS functions associated with synovial inflammation and joint destruction. **Conclusions**: JAKis exert broad effects on RA-FLS function in vitro, likely reflecting their ability to block multiple cytokine-dependent signalling pathways. However, clinical evidence linking these effects to patient outcomes remains limited; further validation is therefore required.

## 1. Introduction

Rheumatoid arthritis (RA) is a chronic, systemic, autoimmune inflammatory disease characterised by persistent synovial inflammation. This inflammation progressively leads to cartilage and bone destruction and loss of joint function [1]. Its aetiopathogenesis is complex and multifactorial, involving interactions among genetic, environmental and immunological factors that trigger a sustained inflammatory response within the synovial tissue [2]. Traditionally, RA has been considered a disease primarily driven by immune cells, such as T and B lymphocytes, macrophages and dendritic cells, as well as by the production of autoantibodies and pro-inflammatory cytokines [3].

However, in recent decades, a significant conceptual shift has occurred in the understanding of RA pathophysiology, with increasing recognition of the central role of resident synovial cells, in particular, fibroblast-like synoviocytes (FLS) [4]. Under physiological conditions, FLS contribute to joint homeostasis through the production of extracellular matrix components and synovial lubricants [5,6]. However, in RA these cells acquire a persistently activated phenotype, characterised by aggressive, proliferative and invasive behaviour that in some respects resembles that of tumour cells [7]. Pathological FLS, also known as RA-FLS, contribute decisively to disease progression through the sustained production of pro-inflammatory cytokines, chemokines and degradative enzymes, such as matrix metalloproteinases (MMPs), which promote the destruction of cartilage and underlying bone [8]. Moreover, these fibroblasts actively interact with immune cells, thereby promoting chronic inflammation within the synovial microenvironment [9].

In this context, the development of therapeutic strategies aimed at modulating these signalling pathways has represented a significant advance in the treatment of RA. Disease-modifying antirheumatic drugs (DMARDs), including conventional synthetic DMARDs (csDMARDs), targeted synthetic DMARDs (tsDMARDs) and biologic DMARDs (bDMARDs), have demonstrated efficacy in controlling inflammation and structural progression [2]. Despite these therapeutic advances, a significant proportion of patients do not achieve an optimal clinical response or experience a loss of efficacy over time [10]. This highlights the need to identify new therapeutic targets and to further elucidate the cellular and molecular mechanisms involved in the disease. In this regard, FLS have emerged as a key therapeutic target because they are not merely passive effectors of inflammation but central players in the perpetuation and amplification of the pathological process.

Therefore, this review analyses the role of FLS as therapeutic targets in RA. It addresses the mechanisms of pathogenic activation underlying their aggressive phenotype and the main immunomodulatory strategies aimed at regulating these cells.

## 2. Materials and Methods

This article presents a traditional narrative review that was not conducted according to a systematic review protocol. Accordingly, no formal systematic screening process or PRISMA-based selection procedure was used, and no meta-analysis or quantitative synthesis was performed. This review provides a comprehensive overview of the current evidence regarding the role of FLS in RA and the mechanisms through which DMARDs modulate FLS behaviour.

The literature search was conducted using PubMed, Scopus, and Google Scholar to identify relevant biomedical and life sciences publications. These sources were selected because PubMed provides extensive coverage of the biomedical literature, Scopus offers broad multidisciplinary indexing and citation tracking, and Google Scholar facilitates the identification of additional relevant studies that may not be captured in conventional bibliographic databases. The most recent literature search was performed in July 2026. The search covered publications from January 2020 to July 2026. Earlier publications were also considered when they constituted seminal contributions or provided essential mechanistic insights into FLS biology, RA pathogenesis, or DMARD mechanisms of action.

Relevant publications were identified using combinations of Medical Subject Headings (MeSH) in PubMed, where applicable, and free-text terms related to the main topics of this review included the following, among others: “rheumatoid arthritis”, “fibroblast-like synoviocytes”, “FLS”, “DMARDs”, “conventional synthetic DMARDs”, “biologic DMARDs”, “targeted synthetic DMARDs”, “methotrexate”, “leflunomide”, “hydroxychloroquine”, “sulfasalazine”, “TNF inhibitors”, “adalimumab”, “infliximab”, “etanercept”, “IL-6 inhibitors”, “tocilizumab”, “abatacept”, “rituximab”, “anakinra”, “JAK inhibitors”, “tofacitinib”, “baricitinib”, “peficitinib”, “filgotinib”, and “upadacitinib”. Search terms were adapted according to the requirements of each search platform and refined during the search process to capture relevant mechanistic studies.

Evidence from both preclinical and clinical studies was considered. Particular emphasis was placed on peer-reviewed original research articles investigating RA-FLS biology, FLS pathogenic functions, and the effects of DMARDs or related immunomodulatory compounds on FLS phenotype and behaviour. Narrative and systematic reviews were also consulted to provide contextual background, identify relevant mechanistic concepts, and facilitate the retrieval of key studies. Studies unrelated to RA-FLS biology, studies lacking mechanistic relevance to DMARD-mediated modulation of FLS, conference abstracts, editorials, letters, preprints, and other non-peer-reviewed sources were not considered. The grey literature was not included.

The authors selected studies based on their relevance to the scope of this narrative review, their contribution to understanding FLS heterogeneity and function, and their ability to provide mechanistic insights into how DMARDs influence processes such as inflammatory mediator production, proliferation, migration, invasion, apoptosis, and intracellular signalling pathways. Studies were selected by the authors according to their relevance to the scope of this narrative review and their contribution to understanding DMARD-mediated modulation of RA-FLS.

**Figure 1:**
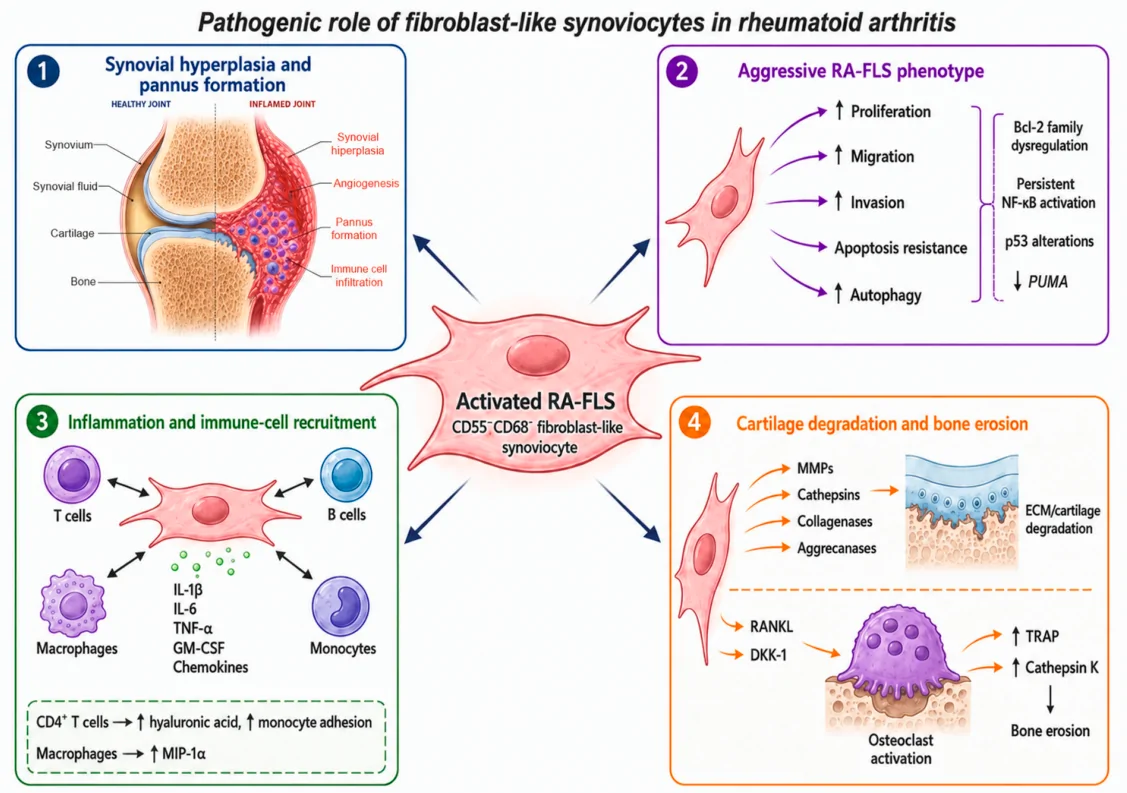
Pathogenic role of fibroblast-like synoviocytes (FLS) in rheumatoid arthritis (RA). In RA, FLS acquire a persistently activated phenotype characterised by increased proliferation, migration, invasion, resistance to apoptosis and enhanced autophagic activity. Upward arrows indicate upregulation or increased expression, whereas downward arrows indicate downregulation or decreased expression. B-cell lymphoma 2 (Bcl-2); Dickkopf-related protein 1 (DKK-1); extracellular matrix (ECM); granulocyte-macrophage colony-stimulating factor (GM-CSF); interleukin (IL); macrophage inflammatory protein (MIP)-1α; matrix metalloproteinase (MMP); nuclear factor kappa B (NF-κB); p53 upregulated modulator of apoptosis (PUMA); receptor activator of nuclear factor kappa-B ligand (RANKL); tumour necrosis factor alpha (TNF-α); and tartrate-resistant acid phosphatase (TRAP). The molecular pathways and cellular processes depicted are based on previously reported mechanisms [1,7,8,16,17,18,20,21,22,23,24,26,27,28,29,30,31,32]. This figure was created using ChatGPT and reviewed by the authors for scientific accuracy.

**Figure 2:**
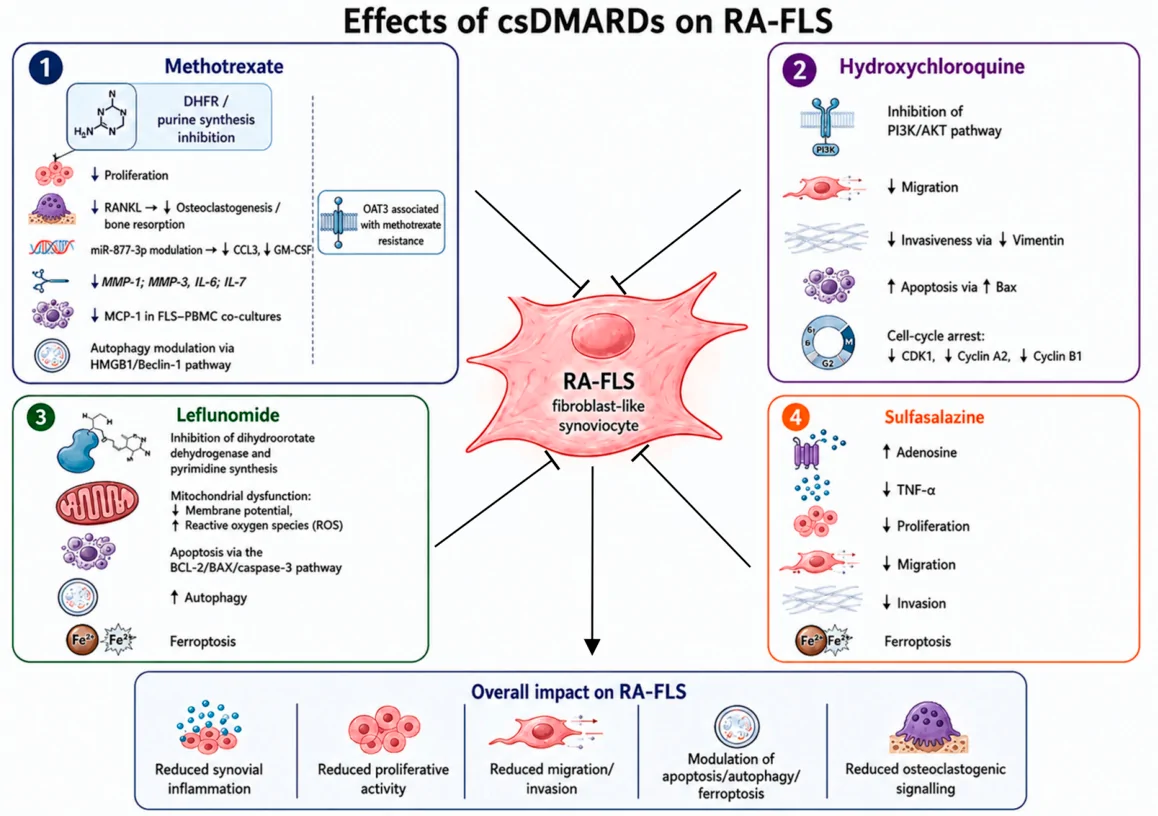
Effects of conventional synthetic DMARDs (csDMARDs) on RA-FLS. csDMARDs, such as methotrexate, hydroxychloroquine, leflunomide and sulfasalazine, modulate several pathogenic functions of RA-FLS. Blunt-ended lines indicate inhibition. The direction of the arrows indicates upregulation/increased expression (upward arrows) or downregulation/decreased expression (downward arrows). Protein kinase B (AKT); BCL2-associated X (BAX); C-C motif chemokine ligand (CCL); cyclin-dependent kinase (CDK); dihydrofolate reductase (DHFR); high-mobility group-box 1 (HMGB1); monocyte chemoattractant protein 1 (MCP-1); organic anion transporter 3 (OAT3); peripheral blood mononuclear cell (PBMC); and phosphatidylinositol 3-kinase (PI3K). The molecular pathways and cellular processes depicted are based on previously reported mechanisms [38,39,40,41,42,43,44,45,46,47,48,49]. This figure was created using ChatGPT and reviewed by the authors for scientific accuracy.

**Figure 3:**
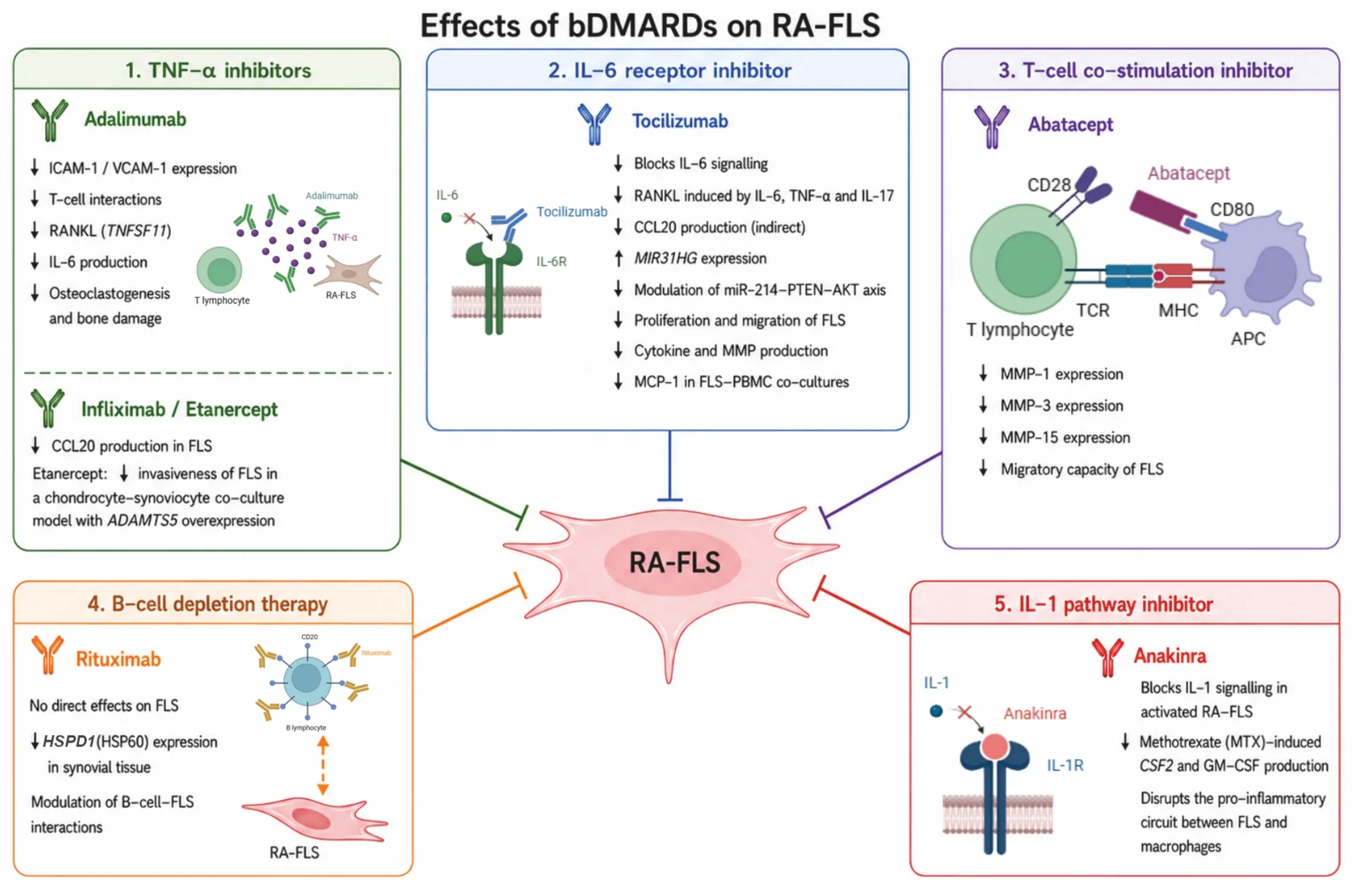
Effects of biologic DMARDs (bDMARDs) on RA-FLS. Different bDMARDs, such as TNF-α inhibitors, IL-6 receptor inhibitors, T-cell co-stimulation inhibitors, B-cell depletion drugs and IL-1 pathway inhibitors, regulate RA-FLS mainly by targeting extracellular cytokine pathways, immune-cell interactions and inflammatory circuits within the synovial microenvironment. Blunt-ended lines indicate inhibition. Dotted arrows indicate molecular interaction or crosstalk. The direction of the arrows indicates upregulation/increased expression (upward arrows) or downregulation/decreased expression (downward arrows). A disintegrin and metalloproteinase with ThromboSpondin motifs 5 (ADAMTS5); heat shock protein 60 (HSP60); HSP family D member 1 (HSPD1); intercellular adhesion molecule 1 (ICAM-1); microRNA 31 host gene (MIR31HG); phosphatase and tensin homologue (PTEN); TNF ligand superfamily member 11 (TNFSF11); and vascular cell adhesion protein 1 (VCAM-1). The molecular pathways and cellular processes depicted are based on previously reported mechanisms [2,29,49,52,55,56,57,58,59]. This figure was created using ChatGPT and reviewed by the authors for scientific accuracy. The diagrams illustrating the mechanisms of action for each drug were created in BioRender. Pascual garcía, S. (2026) https://BioRender.com/cjsnnfm.

During the preparation of this manuscript, the authors used ChatGPT (GPT-5.5, https://chatgpt.com; accessed on 6 July 2026) to improve the language and clarity of the text and used ChatGPT in conjunction with BioRender.com (https://www.biorender.com; accessed on 24 July 2026) to create Figures 1–3. The authors reviewed and edited the output as necessary and take full responsibility for the content of this publication.

## 3. Fibroblast-like Synoviocytes in RA

The synovial membrane is a specialised connective tissue that lines the inner surface of diarthrodial joints, tendon sheaths and bursae [11]. It consists of a superficial intimal layer, composed mainly of FLS and resident macrophages, and a vascularised subintimal layer containing dispersed fibroblasts, adipocytes, blood and lymphatic vessels, as well as a small number of immune cells under physiological conditions [12,13]. Its main functions include lubrication and nutrition of articular cartilage, maintenance of joint homeostasis and regulation of local immune responses [5,6].

FLS are mesenchyme-derived cells characterised by a CD55^+^CD68^−^ phenotype [14]. They are located in both the intimal and subintimal layers of the synovium and perform essential functions in joint homeostasis, including the maintenance of the synovial lining structure and the production of key components of synovial fluid, primarily hyaluronic acid, hyaluronan, lubricin and other extracellular matrix components that contribute to the lubrication and protection of articular surfaces [12,14,15,16,17].

In RA, the synovial membrane undergoes profound structural remodelling characterised by synovial hyperplasia, neoangiogenesis and massive infiltration of immune cells, including T and B lymphocytes, macrophages and plasma cells [16,18]. As a consequence, the synovial lining layer becomes thickened in advanced stages of the disease [11,19]. This process leads to the formation of pannus tissue, a highly invasive inflammatory structure capable of progressively eroding cartilage and subchondral bone [1].

FLS play a central role in this pathological process. During disease progression, they acquire an aggressive phenotype characterised by high proliferative, migratory and invasive capacities and display tumour-like features [7]. This transformation is associated with both excessive proliferation and marked resistance to apoptosis, attributed to alterations in several molecular pathways, including dysregulation of the B-cell lymphoma 2 (Bcl-2) protein family, persistent nuclear factor kappa B (NF-κB) activation, p53 alterations and expression of p53 upregulated modulator of apoptosis (PUMA) [20,21,22]. In parallel, RA-FLS exhibit increased autophagic activity, which may contribute to their survival in the chronic inflammatory environment [18].

RA-FLS actively contribute to joint destruction through the production of degradative enzymes, including MMPs, cathepsins, collagenases and aggrecanases [8]. These molecules degrade essential components of the extracellular matrix and cartilage, promoting tissue invasion and the progression of structural damage [23,24]. In addition, adhesion of RA-FLS to extracellular matrix components, such as collagens and proteoglycans, activates signalling pathways that enhance their invasive behaviour. This phenomenon is reinforced by the overexpression of integrins, particularly those of the β1 family, which increase RA-FLS adhesion and migratory capacity [18].

Beyond their structural role, FLS act as active regulators of the inflammatory microenvironment [25]. These cells produce numerous pro-inflammatory cytokines and chemokines, including interleukin (IL)-1β, IL-6, tumour necrosis factor alpha (TNF-α), granulocyte macrophage colony-stimulating factor (GM-CSF) and various chemotactic mediators, contributing to the recruitment and activation of immune cells within the affected joint [26,27]. They also respond to signals derived from both immune cells and inflammatory metabolites present in the synovium, such as succinate, lactate, eicosanoids and kynurenine, thereby establishing feedback loops that perpetuate chronic inflammation [18].

The bidirectional interaction between FLS and other cellular populations is a key element in RA pathogenesis. RA-FLS do not act solely as structural effector cells but as active regulators of the synovial immune response, coordinating the recruitment, activation and function of T lymphocytes, B cells and macrophages, thereby contributing to the persistence of inflammation [8]. Co-culture of RA-FLS with CD4^+^ T lymphocytes increases hyaluronic acid production and enhances monocyte adhesion, while interaction with macrophages induces the expression of inflammatory mediators such as macrophage inflammatory protein (MIP)-1α [28,29]. Moreover, FLS directly participate in bone remodelling through the production of receptor activator of nuclear factor kappa-B ligand (RANKL), which promotes osteoclast differentiation and activation, and Dickkopf-related protein 1 (DKK-1), which inhibits osteoblastic activity and limits bone repair [30,31]. Indeed, co-cultures of FLS and osteoclasts increase the expression of osteoclastogenic markers such as tartrate-resistant acid phosphatase (TRAP) and cathepsin K, enhancing the bone erosion characteristic of the disease [32].

Overall, current evidence positions FLS as central players in the pathophysiology of RA. These cells not only respond to inflammation but actively contribute to its maintenance through mechanisms such as aberrant proliferation, apoptosis resistance, tissue remodelling, interaction with immune cells and regulation of bone destruction (Figure 1). These characteristics make FLS an attractive therapeutic target for the development of strategies aimed at modifying disease progression and preventing progressive joint damage.

## 4. Effects of DMARDs on RA-FLS

DMARDs constitute the cornerstone of RA therapy and are aimed at achieving sustained disease control and preventing irreversible structural damage. Therapeutic management is based on an early, stepwise and increasingly personalised strategy, as recommended by current clinical guidelines, including those of the American College of Rheumatology (ACR) [33]. Although not all patients progress to advanced erosive disease, early pharmacological intervention has significantly improved long-term functional outcomes.

DMARDs are traditionally classified into three main categories: csDMARDs, tsDMARDs, and bDMARDs. Among csDMARDs, methotrexate is considered the first-line anchor therapy, with leflunomide and sulfasalazine serving as alternative options in cases of contraindication or intolerance. Treatment response is typically evaluated within 3–6 months, after which therapeutic escalation is recommended in patients who do not achieve adequate disease control. This may involve combination therapy or transition to biologic or targeted synthetic agents, depending on clinical response and prognostic factors [33]. bDMARDs are designed to selectively target extracellular mediators of inflammation and include agents directed against TNF-α, such as adalimumab, interleukin-6 receptor (IL-6R) inhibitors such as tocilizumab, and B-cell-depleting therapies such as rituximab [34]. In parallel, tsDMARDs, primarily Janus kinase (JAK) inhibitors (JAKis), act intracellularly by modulating key signalling pathways downstream of multiple cytokine receptors, thereby providing broader control of inflammatory signalling cascades [35].

Importantly, the therapeutic impact of these interventions should not be evaluated solely in terms of systemic immune modulation but also in terms of their ability to modulate FLS behaviour. Although most currently available DMARDs were not originally developed to target FLS specifically, accumulating evidence indicates that they can significantly modulate FLS activation, inflammatory signalling, invasive properties, and other pathogenic functions, thereby contributing to their overall therapeutic effects in RA.

### 4.1. Effect of csDMARDs on RA-FLS

csDMARDs include well-established therapeutic agents such as methotrexate, leflunomide, hydroxychloroquine, and sulfasalazine. Although they have diverse mechanisms of action, their effects converge on reducing synovial inflammation, modulating aberrant immune responses, and preventing structural joint damage (Figure 2). Overall, they act on highly proliferative cells, including lymphocytes and FLS, interfering with metabolic and signalling pathways involved in chronic inflammation [36,37,38].

#### 4.1.1. Methotrexate

Methotrexate is the first-line csDMARD in RA [33,39,50] and acts primarily by inhibiting purine synthesis through blockade of dihydrofolate reductase. This limits FLS proliferation and reduces RANKL expression in these cells, thereby inhibiting osteoclastogenesis and bone resorption [39,40].

In human FLS (hFLS), methotrexate modulates several regulatory mechanisms. These effects include modulation of microRNA-877-3p (miR-877-3p), accompanied by reduced C-C motif chemokine ligand (CCL)3 and GM-CSF production and decreased cellular proliferative capacity [41]. Methotrexate also downregulates pro-inflammatory and degradative mediators such as MMP1, MMP3, IL-6 and IL-7, as well as genes involved in leukocyte migration and tissue homeostasis, such as collagen type XIV alpha 1 chain (*COL14A1*), C-X-C motif chemokine ligand 12 (*CXCL12*) and cytokine-like 1 (*CYTL1*) [42].

In addition, it reduces monocyte chemoattractant protein 1 (MCP-1) production in hFLS–peripheral blood mononuclear cells (PBMCs) co-cultures [43]. At the intracellular level, methotrexate has been reported to modulate autophagy, a process implicated in cell survival and drug resistance, through the high-mobility group-box 1 (HMGB1)/Beclin-1 pathway [44]. Moreover, organic anion transporter 3 (OAT3) has been implicated in mediating methotrexate resistance in hFLS, potentially by regulating intracellular methotrexate accumulation [45].

Interestingly, co-treatment with the black tea-derived compound, theaflavin-3,3′-digallate, has been shown to counteract the pathogenic effects of RA-hFLS by restoring cellular homeostasis through the induction of apoptosis [51]. Furthermore, methotrexate induces transcriptomic reprogramming in RA-hFLS, increasing *IL-1A* and *CSF2* expression [52], thereby reinforcing an IL-1α–*CSF2*/GM-CSF autocrine loop that can be blocked by anakinra [52].

Advanced nanoparticle-based strategies combining methotrexate with small interfering RNA (siRNA) targeting NF-κB and mitogen-activated protein kinases (MAPKs) have been shown to reduce FLS activation and, consequently, the production of TNF-α, IL-1β, IL-6 and MMP-9 [53].

#### 4.1.2. Hydroxychloroquine

Hydroxychloroquine is an antimalarial agent with immunomodulatory activity that acts by interfering with lysosomal function and major histocompatibility complex (MHC)-II-mediated antigen presentation [37]. In FLS, racemic hydroxychloroquine (Rac-HCQ) inhibits the phosphatidylinositol 3-kinase (PI3K)/protein kinase B (AKT) pathway, resulting in reduced migration and invasiveness through downregulation of vimentin expression, together with the induction of apoptosis via increased BCL2-associated X (Bax) expression [46]. Moreover, Rac-HCQ induces cell cycle arrest through downregulation of cyclin-dependent kinase (CDK)1 and cyclins A2 and B1 [46], and has been shown to significantly reduce disease severity in murine models of collagen-induced arthritis (CIA) [46].

#### 4.1.3. Leflunomide

Leflunomide inhibits the mitochondrial enzyme dihydroorotate dehydrogenase, thereby inhibiting pyrimidine synthesis and consequently the proliferation of activated immune cells [47]. In hFLS, it induces mitochondrial dysfunction, characterised by reduced membrane potential and increased production of reactive oxygen species, thereby promoting apoptosis via the BCL-2/BAX/caspase-3 pathway. In addition, it enhances autophagy and reduces joint inflammation in CIA models. Both leflunomide and sulfasalazine have been implicated in the induction of ferroptosis in hFLS [48].

#### 4.1.4. Sulfasalazine

Sulfasalazine exerts anti-inflammatory effects by increasing adenosine levels and reducing TNF-α production, while also inhibiting the proliferation, migration and invasion of hFLS [38,49]. Furthermore, like leflunomide, sulfasalazine has been associated with the induction of ferroptosis in hFLS [48].

### 4.2. Effect of bDMARDs on RA-FLS

bDMARDs exert their therapeutic effects through the selective blockade of soluble mediators and key cellular populations involved in the pathogenesis of RA. This group includes TNF-α inhibitors (infliximab, adalimumab, certolizumab pegol, etanercept and golimumab), IL-6 pathway inhibitors (tocilizumab and sarilumab), T lymphocyte co-stimulation modulators (abatacept), CD20^+^ B-cell-depleting agents (rituximab), and IL-1 inhibitors (anakinra). Collectively, these therapies reduce synovial inflammation, improve clinical symptoms, delay structural progression, and preserve joint function [2,54] (Figure 3).

#### 4.2.1. TNF-α Inhibitors

Adalimumab, a monoclonal anti-TNF-α antibody [2], directly modulates hFLS responses by reducing the TNF-α-induced expression of intercellular adhesion molecule 1 (ICAM-1) and vascular cell adhesion protein 1 (VCAM-1), thereby limiting T-cell interactions and the amplification of synovial inflammation. In addition, it decreases the expression of TNF superfamily member 11 (*TNFSF11*)/RANKL and IL-6 production in response to TNF-α and CXCL10, contributing to reduced osteoclastogenesis and bone damage [29]. Adalimumab has also been reported to modulate multiple soluble mediators in hFLS. Reported changes include reduced levels of transforming growth factor beta (TGF-β), granulocyte colony-stimulating factor (G-CSF), interferon (IFN)γ, IL-10, macrophage colony-stimulating factor (M-CSF) and chemokines such as MIP-1α and MIP-1δ, together with increased lymphotoxin-α and soluble tumour necrosis factor receptor (sTNFR)I/II levels, as well as a sustained reduction in TNF-α following prolonged adalimumab exposure [60].

Infliximab and etanercept also target the TNF-α pathway, reducing CCL20 expression in hFLS [55]. Infliximab is a monoclonal anti-TNF-α antibody, whereas etanercept is a soluble TNF receptor fusion protein [2]. Within this therapeutic axis, novel compounds such as endothelial cell-specific molecule 1 (ESM1)-specific aptamer 04 (ESMA04) have been shown to reduce the pathogenic effects of RA-FLS either as monotherapy or in combination with etanercept [61]. Furthermore, etanercept reverses the increased invasiveness of SW-982 synoviocytes in chondrocyte–synoviocyte interaction models associated with a disintegrin and metalloproteinase with thrombospondin motifs 5 (ADAMTS5) overexpression [49,56].

#### 4.2.2. IL-6 Receptor Inhibitors

Tocilizumab is a monoclonal antibody directed against the IL-6 receptor [2]. In hFLS, it blocks IL-6 signalling and reduces RANKL production induced by IL-6, TNF-α and IL-17 [57], while also indirectly modulating CCL20 expression [55]. At the molecular level, it induces the expression of the long non-coding RNA (lncRNA) microRNA 31 host gene (MIR31HG), which suppresses RA-hFLS proliferation, migration and cytokine and MMP production through modulation of the miR-214–phosphatase and tensin homologue (PTEN)–AKT signalling pathway [58]. In hFLS–PBMCs co-cultures, its effects are associated with reduced production of inflammatory mediators such as MCP-1 [43].

#### 4.2.3. T-Cell Co-Stimulation Inhibitor

Abatacept is a fusion protein composed of the extracellular domain of cytotoxic T-lymphocyte antigen 4 (CTLA-4) linked to the Fc portion of human immunoglobulin (Ig) G1, acting by blocking T-cell co-stimulation through inhibition of the CD80/CD86–CD28 interaction [2]. In hFLS, abatacept administration reduces the expression of MMP-1, MMP-3 and MMP-15, as well as the migratory capacity of these cells, thereby contributing to a reduction in the destructive potential of synovial tissue [2,59].

#### 4.2.4. B-Cell Depletion Therapy

Rituximab is a monoclonal antibody directed against CD20 expressed on B lymphocytes [2]. Although it does not exert direct effects on hFLS, its B-cell-depleting activity is associated with reduced synovial expression of heat shock protein (HSP) family D member 1 (*HSPD1*) which encodes HSP60, an FLS-derived autoantigen implicated in local autoimmunity in RA [62]. This reduction is more pronounced in patients who respond to treatment, suggesting that modulation of B-cell–hFLS interactions contributes to its therapeutic efficacy [62].

#### 4.2.5. IL-1 Pathway Inhibitors

Anakinra is an IL-1 receptor antagonist that blocks IL-1 signalling in activated RA-hFLS. In this context, it inhibits autocrine IL-1 signalling and suppresses methotrexate-induced *CSF2* and GM-CSF expression, thereby disrupting a pro-inflammatory circuit between hFLS and macrophages that is implicated in the persistence of synovitis [52].

### 4.3. Effect of tsDMARDs on RA-FLS

Janus kinase inhibitors (JAKis) are tsDMARD and include tofacitinib, baricitinib, filgotinib, upadacitinib and peficitinib, among others, with differential selectivity profiles for JAK1, JAK2, JAK3 and tyrosine-protein kinase 2 (TYK2) [63]. Collectively, these agents inhibit the Janus kinase–signal transducer and activator of transcription (JAK–STAT) pathway, a central signalling axis for multiple pro-inflammatory cytokines involved in RA pathogenesis, thereby attenuating FLS activation and inflammatory mediator production [2] (Table 1).

JAK inhibitors exert broad modulatory effects on RA-FLS, primarily by interfering with cytokine-dependent signalling pathways involved in inflammation, angiogenesis and tissue remodelling. Tofacitinib, baricitinib, peficitinib, filgotinib and upadacitinib reduce VCAM-1 expression and the production of the pro-angiogenic factor vascular endothelial growth factor (VEGF) in RA-FLS [66,68]. In addition, tofacitinib, baricitinib and peficitinib suppress STAT3 activation induced by multiple cytokines, including IFN-α2b, IFN-γ, IL-6, oncostatin M and leukaemia inhibitory factor (LIF), thereby attenuating cytokine-driven FLS activation and synovial inflammation. Furthermore, tofacitinib and peficitinib reduce the expression of MMP-1 and MMP-3, contributing to the inhibition of extracellular matrix degradation and tissue-destructive responses in RA-FLS [69,71].

Among these agents, tofacitinib has been characterised most extensively; consequently, most of the mechanistic evidence on JAKi-mediated modulation of RA-FLS derives from studies of this compound. Tofacitinib suppresses interferon-induced gene expression [52] and modulates the STAT6/miR-425-5p/insulin-like growth factor 1 (IGF1) axis, resulting in reduced expression of MMP-1, MMP-3, IL-6, TNF-α and IFN-γ [64,65,66,67,68]. It also inhibits IL-6- and IFN-γ-dependent signalling, leading to decreased IL-8, CCL2 and CCL5 expression [69,70,71], reduces MCP-1 production in hFLS–PBMC co-cultures [43], and attenuates the aggressive FLS phenotype by downregulating collagen I and alpha-smooth muscle actin (α-SMA), thereby limiting epithelial-to-mesenchymal transition (EMT)-like processes [72]. At the intracellular level, tofacitinib modulates survival pathways by increasing caspase-3/9 and Bax expression while reducing Bcl-2 and myeloid cell leukaemia 1 (*MCL1)* levels, affecting apoptosis and autophagy [65,71,73]. Furthermore, it promotes dissociation of mitochondrial hexokinase HK2 [74], reduces synovial proliferation and tissue thickening in three-dimensional models, and exhibits tissue-dependent variability in efficacy, with knee-derived hFLS displaying lower sensitivity than hip-derived cells [71,76].

Compared with tofacitinib, evidence for the remaining JAKis is more limited. Baricitinib has been shown to preferentially suppress oncostatin M-induced inflammatory responses, reducing MCP-1, IL-6 and CXCL10 production in hFLS [43,75]. Peficitinib attenuates hFLS activation and synovial membrane thickening and promotes apoptosis through downregulation of the anti-apoptotic genes *BCL-2* and *MCL1* [71]. Current evidence regarding filgotinib and upadacitinib remains limited, with available studies mainly demonstrating inhibition of IL-6-dependent signalling and attenuation of inflammatory pathways in RA-FLS [69].

Overall, although all JAK inhibitors share the ability to suppress pathogenic JAK–STAT signalling in human and murine FLS, direct evidence supporting compound-specific effects remains limited, particularly for newer selective JAK1 inhibitors.

## 5. Comparative Mechanistic Insights into Therapeutic Targeting of FLS

The available evidence suggests that, despite targeting distinct molecular pathways, most DMARDs ultimately converge on the suppression of a limited set of pathogenic RA-FLS functions, including proliferation, inflammatory mediator production, migration, invasion, angiogenesis and osteoclastogenic activity. This observation reinforces the concept that RA-FLS constitute a central effector cell population in RA, integrating inflammatory, tissue-destructive and bone-remodelling signals originating from both innate and adaptive immune responses [8,26,27,30,31]. Notably, the therapeutic efficacy of several DMARD classes appears to be associated with their ability to attenuate the activated RA-FLS phenotype, suggesting that modulation of these stromal cells may represent an important determinant of treatment response.

A comparison of the different DMARD classes reveals clear mechanistic distinctions. csDMARDs predominantly affect intracellular metabolic and survival pathways, thereby limiting RA-FLS proliferation and inflammatory activity. Methotrexate regulates the expression of cytokines, chemokines and matrix-degrading enzymes, reduces MCP-1 and RANKL production, and modulates autophagy-related pathways [40,42,44]. Hydroxychloroquine suppresses PI3K/AKT signalling, reducing FLS migration and invasiveness while inducing apoptosis and cell-cycle arrest [46]. Leflunomide promotes mitochondrial dysfunction, oxidative stress, apoptosis and autophagy, whereas both leflunomide and sulfasalazine have been implicated in the induction of ferroptosis in FLS [48]. In contrast, bDMARDs primarily interfere with extracellular cytokine networks that sustain RA-FLS activation. TNF-α inhibitors reduce adhesion molecule expression, inflammatory cytokine production and osteoclastogenic signalling. Tocilizumab attenuates IL-6-dependent responses, decreases RANKL production and suppresses RA-FLS proliferation, migration and cytokine production through modulation of the MIR31HG/miR-214/PTEN/AKT axis [29,57,58,60]. Abatacept additionally reduces the expression of MMP-1, MMP-3 and MMP-15 and decreases FLS migratory capacity [2,59]. Although these therapeutic classes suppress overlapping pathogenic pathways, they do so through distinct mechanisms reflecting different levels of intervention within the inflammatory cascade.

JAKis occupy an intermediate position between intracellular and extracellular therapeutic strategies. By targeting the JAK–STAT pathway, which integrates signals from multiple cytokines, these agents simultaneously attenuate several pathogenic processes in RA-FLS. Tofacitinib, baricitinib and peficitinib suppress signalling induced by IL-6, interferons, oncostatin M and other inflammatory mediators, resulting in reduced production of MCP-1, IL-6, VEGF, VCAM-1, MMPs and other pro-inflammatory factors [43,68,69,71,75]. Tofacitinib additionally inhibits interferon-regulated gene expression, reduces collagen I and α-SMA expression, modulates autophagy, promotes apoptosis and suppresses inflammatory signalling mediated by IL-6 and IFN-γ [52,65,70,73]. Collectively, these findings indicate that JAK inhibition exerts broad regulatory effects on inflammation, angiogenesis, tissue remodelling and cell survival in RA-FLS.

Another notable observation is that several therapeutically effective agents influence cellular processes extending beyond classical inflammatory signalling. Modulation of apoptosis, autophagy, mitochondrial function and ferroptosis has been reported across multiple DMARD classes [46,48,65,71,73,74], suggesting that metabolic reprogramming represents an important component of RA-FLS pathogenicity. In addition, altered cellular metabolism contributes to the persistence of the aggressive RA-FLS phenotype [9]. Consequently, therapeutic strategies targeting metabolic adaptation may complement cytokine-directed approaches and provide additional opportunities to control synovial pathology.

In addition to currently approved DMARDs, emerging therapeutic approaches may further expand the repertoire of strategies targeting RA-FLS. For example, treatment with pembrolizumab, an anti-programmed death-1 (PD-1) antibody, did not induce MMP-3 production in FLS–PBMCs co-cultures, suggesting that PD-1 pathway blockade does not activate fibroblasts and is unlikely to promote a joint-destructive phenotype in inflammatory arthritis [77]. Similarly, artesunate, an antimalarial agent, has shown potential immunomodulatory effects relevant to RA pathogenesis. In experimental models, artesunate inhibited pyruvate dehydrogenase kinase (PDK)1-induced AKT activation and ribosomal protein S6 kinase A3 (RSK2) phosphorylation, thereby modulating key cellular survival and signalling pathways involved in inflammatory responses [78].

Importantly, the heterogeneity of RA-FLS responses observed across studies suggests that these cells should not be considered a uniform population. Differences in anatomical location and inflammatory context appear to influence drug responsiveness, as illustrated by the lower sensitivity of knee-derived FLS compared with hip-derived FLS to tofacitinib [76]. These observations are supported by recent single-cell RNA sequencing and spatial transcriptomic studies demonstrating that local inflammatory cues and the tissue microenvironment contribute to FLS heterogeneity. These studies have identified functionally distinct fibroblast subsets with predominantly inflammatory, destructive or regulatory phenotypes [25,79,80,81]. Furthermore, circulating pro-inflammatory mesenchymal cells (PRIME cells) have been proposed as a circulating mesenchymal population with phenotypic similarities to synovial subintimal FLS, potentially representing a pre-activated or related state associated with disease flares [24]. Therefore, variability in clinical responses to DMARDs may partially reflect differential effects on specific RA-FLS populations in addition to differences in immune-cell modulation.

Beyond intrinsic fibroblast heterogeneity, patient-specific clinical characteristics may also influence the phenotype and therapeutic responsiveness of RA-FLS. However, most experimental studies provide limited information regarding relevant clinical variables, such as disease activity at the time of sampling, disease duration, previous DMARD exposure, the inflammatory status of the affected joint, or the clinical context in which synovial tissue was obtained (e.g., synovectomy or joint replacement). In many cases, patients were classified according to the 1987 ACR or 2010 ACR/European Alliance of Associations for Rheumatology (EULAR) classification criteria, whereas detailed clinical characterisation is not consistently reported. Consequently, differences in RA-FLS responses across studies may partly reflect variability in disease stage, treatment history or patient characteristics, which should be considered when interpreting the reported effects of DMARDs on fibroblast biology.

The recognition of RA-FLS heterogeneity also has important clinical implications. Persistent activation of pathogenic RA-FLS may also contribute to the development of difficult-to-treat rheumatoid arthritis (D2T-RA) and persistent synovitis despite adequate immune suppression [82,83,84]. Although current DMARDs effectively target major inflammatory pathways, some patients continue to exhibit synovial inflammation and structural progression [10], suggesting that stromal mechanisms may contribute to the persistence of disease despite adequate suppression of immune-mediated inflammation. In this context, RA-FLS may maintain a persistently activated pathogenic phenotype through the sustained production of cytokines, chemokines, matrix-degrading enzymes and osteoclastogenic mediators, thereby perpetuating inflammatory circuits even after immune activation has been partially controlled. Understanding how DMARDs influence these persistent stromal programmes may therefore provide new insights into treatment resistance and support the development of strategies specifically targeting pathogenic fibroblast states in refractory disease.

These concepts also reinforce the potential of fibroblast biology to advance precision medicine in RA. Integrating fibroblast biology into precision medicine strategies may facilitate patient stratification according to dominant pathogenic pathways and support the development of more personalised therapeutic approaches. Such advances could contribute to a shift from broadly suppressing inflammation towards selectively reprogramming the pathogenic stromal compartment that sustains chronic synovial inflammation and joint destruction. In this context, targeted drug-delivery systems, including nanomedicine-based platforms, may enable selective delivery of therapeutic agents to pathogenic FLS populations while minimising systemic exposure and potential adverse effects [85,86,87,88]. Although these approaches remain largely experimental, their combination with current DMARD strategies may enable more precise, cell-specific therapeutic interventions in RA.

Taken together, these findings support a paradigm in which successful DMARD therapy not only suppresses immune activation but also reprogrammes pathogenic stromal cells within the synovial microenvironment. The convergence of multiple therapeutic classes on RA-FLS functions highlights these cells as central mediators of disease persistence and joint destruction. Future therapeutic strategies may therefore benefit from directly targeting RA-FLS-specific pathways involved in survival, metabolic adaptation, migration and tissue invasion, potentially enabling more durable disease control than approaches focused exclusively on immune-cell-derived cytokines.

## 6. Current Limitations of FLS-Targeting DMARDs

Despite the growing body of evidence supporting the modulation of RA-FLS by DMARDs, several limitations hinder definitive conclusions regarding the relative contribution of these effects to clinical efficacy. Most available studies have investigated primary RA-FLS isolated from human synovial tissue, providing valuable insights into fibroblast-specific responses in the context of human disease. However, these studies evaluate DMARD effects under controlled culture conditions, which do not fully reproduce the complexity of the synovial microenvironment, where fibroblasts interact continuously with immune cells, endothelial cells, chondrocytes, extracellular matrix components and mechanical stimuli. Although some studies have incorporated co-culture systems involving FLS and PBMCs or three-dimensional models, these approaches still only partially reproduce the cellular interactions and spatial organisation occurring in vivo [43,46,48,56,71].

Second, considerable methodological heterogeneity exists across studies, including differences in cell source, anatomical location, culture conditions, stimulation protocols and drug concentrations, making direct comparisons challenging. This variability may significantly influence treatment responses, as illustrated by the observation that knee-derived RA-FLS display lower sensitivity to tofacitinib than hip-derived RA-FLS [76].

Third, many investigations focus on a limited number of outcomes, such as cytokine production, proliferation or apoptosis, whereas integrated analyses simultaneously evaluating inflammatory, metabolic, angiogenic and tissue-remodelling pathways remain scarce. Furthermore, most studies evaluate the effects of individual DMARDs in isolation, thereby limiting potential interactions between signalling pathways and therapeutic mechanisms.

Another factor that should be considered when comparing the effects of different DMARD classes is the unequal distribution of available evidence across therapeutic agents. The apparent magnitude or consistency of reported effects for specific drugs may partly reflect the larger number of published studies available for those treatments rather than a true difference in biological activity on RA-FLS. Consequently, comparative interpretations should be made cautiously, as some DMARDs remain less extensively investigated, and their potential effects on fibroblast biology may be underestimated due to limited experimental evidence.

Another important limitation is the limited consideration of RA-FLS heterogeneity. Recent single-cell studies have identified multiple fibroblast subsets with distinct inflammatory, destructive or regulatory functions [11,79,80], yet most experimental studies continue to analyse mixed fibroblast populations, potentially masking subset-specific drug responses.

Finally, although numerous studies demonstrate direct effects of DMARDs on RA-FLS, the extent to which these cellular changes contribute to clinical responses remains largely uncertain, as mechanistic studies linking fibroblast modulation to patient outcomes are still limited. Consequently, current evidence should be interpreted with caution, and further translational studies are required to establish the clinical relevance of RA-FLS-targeted effects.

## 7. Conclusions

Collectively, the available evidence indicates that RA-FLS are not merely passive targets of DMARD therapy but represent central orchestrators of synovial inflammation, tissue destruction and disease persistence. Despite acting through distinct molecular mechanisms, csDMARDs, bDMARDs and JAKis converge on the modulation of key pathogenic RA-FLS functions, suggesting that fibroblast reprogramming may constitute an important component of therapeutic efficacy. Among the DMARD classes evaluated, JAKis appear to exert broad modulatory effects on RA-FLS biology by simultaneously targeting multiple intracellular signalling pathways. However, this observation is mainly supported by in vitro studies, and direct comparative analyses between different DMARD classes remain limited. No current therapeutic strategy fully addresses the cellular heterogeneity and functional complexity of RA-FLS within the synovial microenvironment. Future studies should therefore focus on defining fibroblast subset-specific signatures, identifying predictive biomarkers of treatment response and developing therapies that directly target pathogenic fibroblast populations. Such approaches may facilitate the transition towards precision medicine strategies capable of achieving more sustained disease control and preventing structural joint damage.

## Figures and Tables

**Table 1:** Comparative summary of Janus kinase inhibitor (JAKi) selectivity profiles and effects on RA-FLS.

JAKis	JAK Selectivity Profile	Key Differential Effects on FLS Functions	Distinctive Molecular Features	References
Tofacitinib	JAK1/3 and partial JAK2 inhibitor	Reduces VCAM-1, VEGF, MMP-1, MMP-3, IL-6, IL-8, CCL-2, CCL-5, TNF-α, and IFN-γ expression. Decreases MCP-1 production, and EMT-like processes. Modulates autophagy and apoptosis and dissociation of HK2.	Suppresses the expression of interferon-induced genes. Modulates STAT6/miR-425-5p/IGF1 axis. Suppresses STAT3 activation induced by cytokines. Downregulates collagen I and α-SMA. Increases caspase-3/9 and Bax and reduces Bcl-2 and *MCL1* expression.	[43,52,63,64,65,66,67,68,69,70,71,72,73,74]
Baricitinib	Selective JAK1/2 inhibitor	Reduces VCAM-1 and VEGF expression, and MCP-1, IL-6 and CXCL10 production.	Suppresses STAT3 activation induced by cytokines.	[43,63,66,68,69,71,75]
Peficitinib	pan-JAK inhibitor	Suppresses VCAM-1, VEGF, MMP-1, and MMP-3 expression. Promotes apoptosis.	Suppresses STAT3 activation induced by cytokines. Inhibits *BCL2* and *MCL1* anti-apoptotic genes.	[63,66,68,69,71]
Filgotinib	Selective JAK1 inhibitor	Reduces VCAM-1 and VEGF expression.	Inhibits IL-6 induced JAK-STAT pathway.	[63,66,68,69]
Upadacitinib	Selective JAK1 inhibitor	Reduces VCAM-1 and VEGF expression.	Inhibits IL-6 induced JAK-STAT pathway.	[63,66,68,69]

This table summarises the selectivity profiles of currently available JAK inhibitors, their principal effects on RA-FLS functions and their distinctive molecular mechanisms. Alpha-smooth muscle actin (α-SMA); C-X-C motif chemokine ligand (CXCL); epithelial-to-mesenchymal transition (EMT); hexokinase 2 (HK2); insulin-like growth factor 1 (IGF1); myeloid cell leukaemia 1 (MCL1); and signal transducer and activator of transcription (STAT).

## Data Availability

No new data were created or analyzed in this study. Data sharing is not applicable to this article.

## Abbreviations

The following abbreviations are used in this manuscript:

α-SMA	Alpha-smooth muscle actin
ACR	American college of rheumatology
ADAMTS5	A disintegrin and metalloproteinase with ThromboSpondin motifs 5
AKT	Protein kinase B
Bax	BCL2-associated X
Bcl-2	B-cell lymphoma 2
bDMARDs	Biologic DMARDs
CCL	C-C motif chemokine ligand
CDK	Cyclin-dependent kinase
CIA	Collagen-induced arthritis
*COL14A1*	Collagen type XIV alpha 1 chain
csDMARDs	Conventional synthetic DMARDs
CTLA-4	Cytotoxic T-lymphocyte antigen 4
CXCL	C-X-C motif chemokine ligand
*CYTL1*	Cytokine-like 1
D2T-RA	Difficult-to-treat rheumatoid arthritis
DHFR	Dihydrofolate reductase
DKK-1	Dickkopf-related protein 1
DMARDs	Disease-modifying antirheumatic drugs
ECM	Extracellular matrix
EMT	Epithelial-to-mesenchymal transition
ESMA	Endothelial cell-specific molecule 1 (ESM1)-specific aptamer
EULAR	European Alliance of Associations for Rheumatology
FLS	Fibroblast-like synoviocytes
G-CSF	Granulocyte colony-stimulating factor
GM-CSF	Granulocyte macrophage colony-stimulating factor
hFLS	Human FLS
HK2	Hexokinase 2
HMGB1	High-mobility group-box 1
HSP60	Heat shock protein 60
HSPD1	HSP family D member 1
ICAM-1	Intercellular adhesion molecule 1
IFN	Interferon
Ig	Immunoglobulin
IGF1	Insulin-like growth factor 1
IL	Interleukin
IL-6R	Interleukin 6 receptor
JAK	Janus kinase
JAKis	JAK inhibitors
LIF	Leukaemia inhibitory factor
lncRNA	Long non-coding RNA
MAPK	Mitogen-activated protein kinases
MCL1	Myeloid cell leukaemia 1
MCP-1	Monocyte chemoattractant protein 1
M-CSF	Macrophage colony-stimulating factor
MeSH	Medical subject headings
MHC	Major histocompatibility complex
MIP	Macrophage inflammatory protein
miR	MicroRNA
MIR31HG	MicroRNA 31 host gene
MMP	Matrix metalloproteinase
NF-κB	Nuclear factor kappa B
OAT3	Organic anion transporter 3
PBMC	Peripheral blood mononuclear cell
PD-1	Programmed death-1
PDK	Pyruvate dehydrogenase kinase
PI3K	Phosphatidylinositol 3-kinase
PRIME	Pro-inflammatory mesenchymal cells
PTEN	Phosphatase and tensin homolog
PUMA	p53 upregulated modulator of apoptosis
RA	Rheumatoid arthritis
Rac-HCQ	Racemic hydroxychloroquine
RANKL	Receptor activator of nuclear factor kappa-B ligand
RSK2	Ribosomal protein S6 kinase A3a
siRNA	Small interfering RNA
STAT	Signal transducer and activator of transcription
sTNFR	Soluble tumour necrosis factor receptor
TGF-β	Transforming growth factor beta
TNF-α	Tumour necrosis factor alpha
TNFSF11	TNF ligand superfamily member 11
TRAP	Tartrate-resistant acid phosphatase
tsDMARDs	Targeted synthetic DMARDs
TYK2	Tyrosine-protein kinase 2
VCAM-1	Vascular cell adhesion protein 1
VEGF	Vascular endothelial growth factor
